# FEAR OF FALLING PREDICTS NEGATIVE PERCEPTIONS OF AGING AMONG LOW-INCOME COMMUNITY-DWELLING OLDER ADULTS

**DOI:** 10.1093/geroni/igad104.3350

**Published:** 2023-12-21

**Authors:** Evette Trahan, Renata Komalasari, Amber Blount, Nichole Lighthall, Ladda Thiamwong

**Affiliations:** University of Central Florida, Orlando, Florida, United States; Penn State Nursing, State College, Pennsylvania, United States; University of Central Florida, Orlando, Florida, United States; University of Central Florida, Orlando, Florida, United States; University of Central Florida, Orlando, Florida, United States

## Abstract

Negative self-perceptions of aging (NSPA) are associated with various health-related outcomes, including fear of falling (FOF). Despite the leading cause of injury, disability, and hospitalization among racially diverse low-income older adults, FOF’s predictive potential effect on NSPA is less studied. This was a cross-sectional study with a sampling of 54 independent and cognitively healthy older adults (4 males, 47 females, mean age 73.91, SD: 6.4; 61-87 years old) in a low-income community. A five-item NSPA Likert scale, part of the Brief Ageing Perceptions Questionnaire (B-APQ), was used to assess the negative self-perceptions of aging, with higher scores indicating fewer negative views. The Short Fall Efficacy Scale International questionnaire was used to evaluate the level of concern about falls for a range of activities of daily living. More than half of the participants were African Americans (27 or 50%), completed high school (29 or 53.7%), lived alone (37 or 68.5%), and earned just enough financially (29 or 53.7%). The simple regression results indicated that the model explained 14.9% of the variance (R2=.149, F(1,52) = 9.13, p =.004). Fear of falling (FOF) (β = -.406, p =.004) was negatively associated with NSPA. As FOF scores increased, NSPA scores decreased, signifying higher FOF, accentuating worse perceived aging. Future longitudinal research should focus on the relationship between FOF and NSPA in increasing fall risk and injurious falls, especially in ethnically diverse low-income older adults, to further clarify this relationship and its consequences. Additionally, assessing FOF and providing interventions may enhance positive perceptions of aging.

